# Stereotactic body radiotherapy for chronic obstructive pulmonary disease patients undergoing or eligible for long-term domiciliary oxygen therapy

**DOI:** 10.1093/jrr/rrv064

**Published:** 2015-10-20

**Authors:** Yu Hara, Atsuya Takeda, Takahisa Eriguchi, Naoko Sanuki, Yousuke Aoki, Shuichi Nishimura, Tatsuji Enomoto, Masaharu Shinkai, Akihiko Kawana, Takeshi Kaneko

**Affiliations:** 1 Respiratory Disease Center, Yokohama City University Medical Center, Yokohama, Kanagawa, Japan; 2 Radiation Oncology Center, Ofuna Chuo Hospital, Kamakura, Kanagawa, Japan; 3 Department of Infectious Diseases and Pulmonary Medicine, National Defense Medical College, Tokorozawa, Saitama, Japan; 4 Department of Respiratory Medicine, Ofuna Chuo Hospital, Kamakura, Kanagawa, Japan; 5 Department of Pulmonology, Yokohama City University Graduate School of Medicine, Kanazawa, Kanagawa, Japan

**Keywords:** chronic obstructive pulmonary disease, interstitial pneumonia, long-term domiciliary oxygen therapy, lung cancer, radiation pneumonitis, stereotactic body radiotherapy

## Abstract

A major cause of death in patients undergoing long-term domiciliary oxygen therapy (LTOT) is lung cancer progression. In our institution, we actively perform stereotactic body radiotherapy (SBRT) on patients with early-stage non–small-cell lung cancer undergoing LTOT. In this study, we retrospectively analyzed the treatment efficacy and safety of SBRT for patients with T1-3N0M0 non–small-cell lung cancer who had been prescribed LTOT for treatment of chronic obstructive pulmonary disease (COPD). A total of 24 patients were studied. Their median age was 74 years (range, 63–87 years). The median duration from the start of LTOT to SBRT was 23 months (range, 0–85 months). Four of the 24 patients underwent lobectomy due to lung cancer. The median follow-up duration was 29 months (range, 5–79 months). One patient had a local recurrence. The median survival time was 30 months. The 3-year overall survival was 49%. In 6 of the 24 patients (25%), COPD presented with interstitial pneumonia. The 3-year overall survival for patients with COPD without interstitial pneumonia was significantly better than that for patients with both COPD and interstitial pneumonia (67% and 0%, respectively; *P* < 0.0001). Grade 5 radiation pneumonitis occurred in one patient (4%) with COPD with interstitial pneumonia. SBRT was tolerated by patients with early-stage non–small-cell lung cancer undergoing LTOT. SBRT should be considered for patients undergoing LTOT. However, clinicians should consider the risk of severe radiation pneumonitis in patients with interstitial pneumonia.

## INTRODUCTION

The number of patients receiving long-term domiciliary oxygen therapy (LTOT) has progressively increased [1]. According to the 2010 published data from *The Japanese White Paper on Home Respiratory Care* [2], the most common pulmonary diseases among patients undergoing LTOT are chronic obstructive lung disease (COPD) (45%), pulmonary fibrosis (18%), pulmonary tuberculosis sequelae (12%) and lung cancer (6%). One of the major causes of death in patients undergoing LTOT is lung cancer progression [3]. Surgical treatment is often contraindicated for early-stage lung cancer. Even if this treatment can be performed, postsurgical complications of lung cancer resection in patients with COPD or severe chronic ventilatory impairment are frequent, and the postoperative decline in pulmonary function is not negligible [4, 5]. Stereotactic body radiation therapy (SBRT) is associated with minimal morbidity and high local control rates comparable with those of lobectomy and has thus become the standard treatment option for inoperable early-stage lung cancer [6, 7]. Furthermore, it has been previously reported that SBRT has a limited effect on long-term pulmonary function decline, especially in patients with severe COPD [8].

In this study, we retrospectively analyzed 24 patients who were prescribed LTOT while undergoing SBRT due to T1-3N0M0 non–small-cell lung cancer (NSCLC) and evaluated the efficacy and safety of this treatment, including the decline in lung function and frequency of radiation pneumonitis (RP).

## MATERIALS AND METHODS

### Study location and patients

We retrospectively identified consecutive patients with T1-3N0M0 NSCLC treated with SBRT and undergoing LTOT in our institution from 2006 to 2013. LTOT was prescribed at each patient's referral hospital. The LTOT criteria were based on the guidelines of the Japan Respiratory Society [1, 9] and were as follows: (i) PaO_2_ of ≤55 mmHg in ambient air at rest or (ii) PaO_2_ of ≤60 mmHg in ambient air at rest in the presence of pulmonary hypertension or with severe hypoxemia during exercise or sleep. However, patients with PaO_2_ of >60 mmHg at rest but severe hypoxemia during exercise or sleep were prescribed LTOT when the physician considered this therapy appropriate. All patients provided written informed consent for inclusion in this study. Our institution review board approved this study (No. 2012–002).

### SBRT

SBRT methods have been previously described [10, 11]. Briefly, long-scan-time computed tomography (CT) was used to directly visualize the internal target volume after immobilizing the patient with a vacuum pillow [10]. The planning target volume was determined by adding a margin of 6–8 mm to the internal target volume. Dynamic conformal multiple arc irradiation was used for SBRT. SBRT was performed with 40–60 Gy in five fractions prescribed to the 80% isodose line of the maximum dose. For the patients with interstitial pneumonia (IP), SBRT was performed without any specific modifications to the methods (doses administered, fractionation, beam arrangements, respiratory movement control, etc.).

We carefully informed all patients (especially those presenting with IP) of the necessity for SBRT and the risk of deterioration of pulmonary function and RP or acute exacerbation of IP.

### Follow-up and analysis of chest CT and pulmonary function

For all patients, follow-up CT was performed before SBRT, at 1 and 3 months after SBRT, and at 3-month intervals during the first 2 years. Follow-up CT was then performed at 4- to 6-month intervals. CT findings were reviewed by two pulmonologists and one radiologist. The diagnostic process of IP was based on the American Thoracic Society (ATS)/European Respiratory Society (ERS) international multidisciplinary consensus classification of idiopathic interstitial pneumonias (IIPs) [12]. Also, we carefully assessed the underlying conditions (such as collagen vascular diseases) and environmental exposures (including drug use and occupational exposures).

For all patients, pulmonary function test (PFT) parameters before SBRT were measured according to the ATS guidelines [13], using a pulmonary function instrument with computer processing (Spiro Shift SP-750; Fukuda Denshi Co., Tokyo, Japan). The parameters assessed included the vital capacity (VC), forced VC, and forced expiratory volume in 1 s (FEV_1.0_). The predicted FEV_1.0_ and VC were calculated according to the standard values of the Global Initiative for Chronic Obstructive Lung Disease (GOLD), and the patients with COPD were diagnosed according to the GOLD criteria [14]. We also repeatedly measured the PFT parameters after SBRT and compared the pre- and post-SBRT PFT parameters to assess the decline in lung function after SBRT. Post-SBRT PFT was performed >10 months after SBRT.

### Statistical analysis

Survival curves were prepared using the Kaplan–Meier method, and the curves were compared using the log-rank test. We also evaluated the local control rate. Local control was defined as free of local progression that was 1.2 or more times the dimensions of original tumor. The Wilcoxon matched-pair signed-rank test was performed to compare the pre- and post-SBRT PFT parameters. *P* values of <0.05 were considered statistically significant. All statistical analyses were performed using JMP10 software (SAS Institute Inc. NC, USA).

## RESULTS

### Patient characteristics

In our institution from 2006 to 2013, 24 patients with T1-3N0M0 NSCLC undergoing or eligible for LTOT were treated with SBRT. Three of these 24 patients met the LTOT criteria and were recommended to undergo LTOT, but they refused to undergo LTOT before SBRT. However, they agreed to undergo LTOT after SBRT after strong recommendations by their physicians. The baseline characteristics of these patients are listed in Table 1. The median age of the treated patients was 74 years (range, 63–87 years). All patients had a Karnofsky performance status of 1. The reasons for performing LTOT were COPD in 13 patients, and COPD with other pulmonary disorders in 11 patients These other pulmonary disorders consisted of bronchial asthma in 3 patients, chronic pulmonary thromboembolism in 1, IP in 3, post-lobectomy due to lung cancer in 1, and both IP and post-lobectomy in 3. The subclassifications of IP were idiopathic pulmonary fibrosis (IPF) in 1 patient, IIP other than IPF in 3 patients, pneumoconiosis in 1 patient and asbestos-related disease in 1 patient (serum lactate dehydrogenase, 241 IU/l (range, 166–227 IU/l); serum surfactant protein D, 97.4 ng/ml (range, 72–237 ng/ml); serum sialylated carbohydrate antigen, 509 U/ml (range, 362–1506 U/ml)). The median duration of LTOT between LTOT initiation and SBRT was 23 months (range, 0–85 months), and the median oxygen flow was 1 l/min (range, 0–2 l/min) at rest and 1.5 l/min (range, 0–4 l/min) during exercise.

**Table 1.** Patient characteristics

Characteristics	
Total patients, *n*	24
Age, years	74 (63–87)
Sex, male (female)	22 (2)
Body mass index	22.8 (14.8–29.4)
Brinkman index	1200 (350–4000)
Karnofsky Performance Status (0/1/2)	0/24/0
Causes of LTOT	
COPD alone	13
COPD with asthma	3
COPD with chronic pulmonary thromboembolism	1
COPD post lobectomy	1
COPD with IP	3
COPD with IP and post lobectomy	3
GOLD criteria (I/II/III/IV)	4/6/7/7
Oxygen flow, l/minute	Rest: 1 (0–2) Exercise: 1.5 (0–4)
Duration of LTOT between LTOT initiation and SBRT, months	23 (0–85)
Serum lactate dehydrogenase, IU/l	215 (144–268)
Serum surfactant protein D, ng/ml (22 patients measured)	77.5 (20–401)
Serum sialylated carbohydrate antigen, U/ml (23 patients measured)	404 (157–1506)
PFT	
VC, l	2.47 (1.35–3.57)
VC, %predicted	73.0 (44.9–109.7)
FEV_1.0_, l	1.12 (0.45–2.16)
FEV_1.0_, %predicted	42.9 (19.2–92.0)
Histology	
Adenocarcinoma	7
Squamous cell carcinoma	8
Large cell carcinoma	1
Unclassified non-small-cell lung cancer	1
Histologically unproven	7
T stage (N0M0): T1/T2/T3	14/9/1
Location: peripheral/central	18/6
Maximum diameter, mm	27 (16–53)
Internal target volume, ml	32.3 (2.8–37.7)
Planning target volume, ml	85.4 (18.1–116)
Normal lung volume receiving ≥20 Gy, %	3.5 (1.2–7.8)
Mean lung dose, cGy (range)	332 (164–511)
Total dose: 60/50/45/40 Gy	1/15/1/7
Radiation pneumonitis Grade 0–1/2/3/4/5	8/3/0/0/1
SBRT follow-up PFT, months	14 (10–28)

All data are given as median (range) unless otherwise indicated. COPD = chronic obstructive pulmonary disease, FEV_1.0_ = forced expiratory volume in 1 s, GOLD = Global Initiative for Chronic Obstructive Lung Disease, IP = interstitial pneumonia, LTOT = long-term domiciliary oxygen therapy, PFT = pulmonary function test, SBRT = stereotactic body radiotherapy, VC = vital capacity.

### Survival

**Fig. 1.**
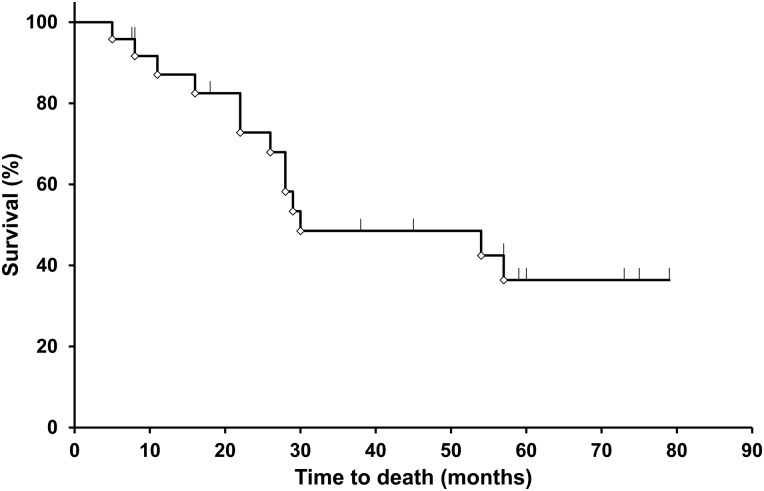
Time to death of all causes of COPD after stereotactic body radiotherapy. The median survival time in 24 patients was 30 months. The 1-, 3- and 5-year overall survival figures were 87%, 49% and 36%, respectively.

**Fig. 2.**
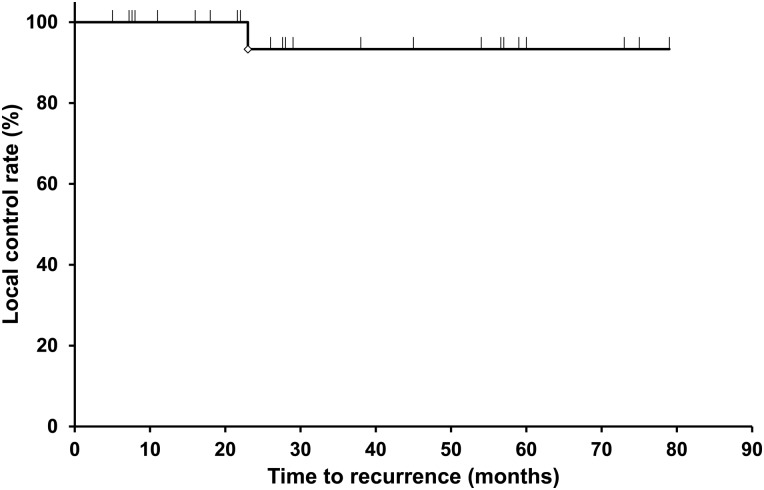
Local control rate of all causes of COPD after stereotactic body radiotherapy. Only one patient had a local recurrence 23 months after SBRT. The 1-, 3- and 5-year local control rates were 100%, 93% and 93%, respectively.

Table 2 and Fig. 1 show the overall survival (OS) in the 24 patients. Four patients (17%) died of lung cancer. The median follow-up duration was 29 months (range, 5–79 months), and the median survival time was 30 months. The 1-, 3- and 5-year OS were 87%, 49% and 36%, respectively. One patient had a local recurrence 23 months after SBRT. The 1-, 3- and 5-year local control rates were 100%, 93% and 93%, respectively (Table 2, Fig. 2).

**Table 2.** Length of follow-up, local control, and survival after stereotactic body radiotherapy

Parameters	
Follow-up, months	29 (5–79)
Follow-up, number	24
Median overall survival, months	30
Percent surviving	
1 year, %	87
3 years, %	49
5 years, %	36
Local control rate, %	Recurrence: 1 of 24 patients
1 year, %	100
3 years, %	93
5 years, %	93

All data given as median (range) unless otherwise indicated.

**Fig. 3.**
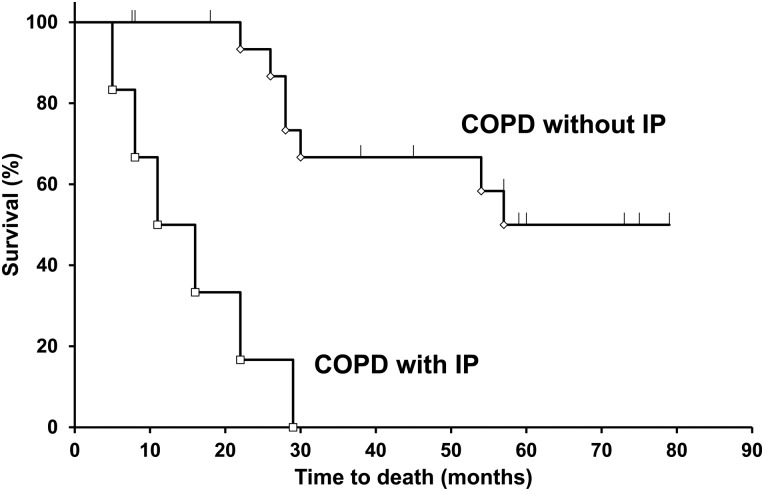
Comparisons between patients with COPD without interstitial pneumonia (IP) and those with COPD with IP. The 18 patients were the ‘COPD without IP’ group and the 6 patients were the ‘COPD with IP’ group. The 3-year overall survival in patients with COPD without IP was significantly better than that in patients with COPD with IP (67% and 0%, respectively; *P* < 0.0001).

The 24 patients were divided into two groups (COPD without IP, *n* = 18; COPD with IP, *n* = 6). We evaluated the Kaplan–Meier curves of the two groups and found significant differences using the log-rank test (*P* < 0.0001) (Fig. 3). The 1-, 3- and 5-year OS of patients with COPD without IP were 100%, 67% and 50%, and those of patients with COPD with IP were 50%, 0% and 0%, respectively.

### Pulmonary function decline and RP after SBRT

We followed up 16 patients with PFT. Eight patients could not be followed up with PFT because of loss to follow-up (*n* = 4) or an inability to perform measurements because of extrapulmonary complications (*n* = 1) or death (tumor progression, *n* = 2; RP, *n* = 1). The median follow-up duration (from SBRT to PFT) was 14 months (range, 10–28 months). The median pre-SBRT FEV_1.0_, post-SBRT FEV_1.0_, and difference between the two according to GOLD criteria are shown in Table 3. There were no significant differences between the pre- and post-SBRT PFT data in GOLD I, II group, GOLD III, IV group and overall cases.

**Table 3.** Variations of pulmonary function tests according to GOLD criteria

GOLD criteria	Parameters	Pre-SBRT	Post-SBRT	Pre-Post	*P* values Pre vs. Post
Mild to moderate COPD (I, II) *n* = 6	FEV_1.0_, L	1.40 (0.85–2.16)	1.30 (0.61–2.24)	0.14 (−0.08–0.38)	0.15
	FEV_1.0_ %predicted	61.2 (50.2–89.4)	55.6 (29.4–95.0)	5.5 (−5.7–22.2)	0.38
	VC, L	2.24 (1.73–3.37)	2.23 (1.33–3.12)	0.16 (−0.09–0.47)	0.11
	VC %predicted	79.6 (52.1–105.1)	76.5 (46.5–104.1)	3.0 (−6.3–17.2)	0.29
Severe COPD (III, IV) *n* = 10	FEV_1.0_, L	0.92 (0.58–1.30)	0.88 (0.47–1.39)	0.02 (−0.16–0.44)	0.45
	FEV_1.0_ %predicted	36.0 (19.3–44.1)	33.5 (21.2–48.1)	2.4 (−6.2–7.9)	0.80
	VC, L	2.68 (1.72–3.57)	2.71 (1.59–3.76)	−0.07 (−0.31–0.78)	0.94
	VC %predicted	73.9 (49.4–109.7)	76.0 (51.5–117.7)	−2.0 (−9.7–22.1)	0.70
Overall cases *n* = 16	FEV_1.0_, L	1.12 (0.58–2.16)	1.02 (0.47–2.24)	0.10 (−0.16–0.44)	0.11
	FEV_1.0_ %predicted	42.9 (19.3–89.4)	42.9 (21.2–95.0)	<0.01 (−6.2–22.2)	0.47
	VC, L	2.62 (1.72–3.57)	2.68 (1.33–3.76)	−0.07 (−0.31–0.78)	0.33
	VC %predicted	77.0 (49.4–109.7)	76.0 (46.5–117.7)	1.04 (−9.7–22.1)	0.70

All data given as median (range) unless otherwise indicated. COPD = chronic obstructive pulmonary disease, FEV_1.0_ = forced expiratory volume in 1 s, GOLD = global Initiative for Chronic Obstructive Lung Disease, SBRT = stereotactic body radiotherapy, VC = vital capacity.

Twelve patients (50%) presented with RP; eight patients (33%) had Grade 1 RP, three patients (13%) had Grade 2 RP, and one patient (4%) had Grade 5 RP. The patient with Grade 5 RP was a 72-year-old man and a previous smoker. Previously, he had been treated with left lower lobectomy due to a pulmonary nodule that proved to be benign and that was suspected to be round atelectasis. He was then diagnosed with asbestos-related disease due to the presence of an asbestos body in a lung specimen and exhibited subclinical IP on high-resolution CT. Thirteen years later he underwent SBRT (40 Gy/5 fractions) to treat squamous cell lung carcinoma in the right lung (T2aN0M0). Two months after SBRT, he developed an onset of RP. And he died despite steroid therapy eleven months after presenting RP.

## DISCUSSION

SBRT has recently become the standard treatment option for inoperable early-stage lung cancer because of minimal morbidity and high local control rates comparable with those of lobectomy [6]. In a Phase II study, patients who underwent SBRT had a survival rate of 55.8% at 3 years and a median survival time of 48.1 months [7]. Stephan *et al.* [15] reported that even in patients with severe ventilatory impairment, SBRT was associated with a 0% 30-day mortality rate after treatment and no Grade ≥3 RP. Furthermore, in previously reported data of patients with severe COPD, only small and non-significant declines in FEV_1.0_ and forced VC were seen 1 year after SBRT [8]. Therefore, SBRT could become the main therapeutic option for patients with early-stage lung cancer and severe pulmonary function decline. However, there are almost no studies on the efficacy and safety of SBRT in patients undergoing LTOT for COPD.

Patients with COPD who underwent LTOT historically had a poor prognosis. The 3- and 5-year OS rates of these patients were 51% and 22%, respectively, and the median survival was 3.0 years [1]. Patients with untreated clinical Stage I NSCLC also had a poor prognosis. The 5-year OS rate of these patients was 14–24%, and the median survival time was 17–27 months [16]. Therefore, both patients with early lung cancer and those undergoing LTOT seemed to have poor prognosis. However, compared with these studies, we obtained good outcomes of SBRT for these patients. The 1-, 3- and 5-year OS were 87%, 49% and 36%, respectively, and the median survival time was 30 months. These results indicate that SBRT may be more beneficial for patients with lung cancer undergoing LTOT than those undergoing no specific treatment (natural course). Furthermore, our current study results are not inferior to those of patients with Stage I NSCLC and poor ventilatory function; the actuarial OS was 79–95% at 1 year and 43–70% at 3 years [4].

Lung cancer surgery in patients with poor ventilatory function remains controversial because of severe postoperative complications. Lau *et al.* [17] reported that of patients with poor ventilatory function (as defined by a predicted postoperative FEV_1.0_ of <40%), 11% died in hospital, 11% of patients developed respiratory complications, and 14% developed non-respiratory complications. However, compared with that study, our present study on SBRT revealed a 0% 30-day mortality rate; other studies on SBRT have obtained similar results [4, 15]. Furthermore, in our present study, there were no significant differences between the pre- and post-SBRT PFT data, regardless of GOLD criteria, although we were only able to analyze the variations in pulmonary function 1 year after SBRT in 16 patients. Also, Yoshitake *et al.* [18] reported that in 15 Stage I NSCLC patients requiring LTOT, the oxygen flow required increased slightly at follow-up periods greater than one year after SBRT, but was still at acceptable levels. Therefore, a remarkable decline in pulmonary function did not seem to occur, even in patients with poor ventilatory function undergoing LTOT.

The combination of COPD and IP is suspected to be a risk factor for critical RP after SBRT and unsatisfactory OS. In our present study, the 1-, 3- and 5-year OS rates in patients with lung cancer with complications of COPD and IP were 50%, 0% and 0%, respectively, which were worse than those in patients with COPD without IP. Additionally, 1 of the 24 patients (4%) developed Grade 5 RP. Historically, IP has been suspected to be a risk factor for severe RP. Yamaguchi *et al.* [19] reported that 1 of 16 patients (6.2%) with the presence of subclinical IP in the pre-SBRT CT findings presented with Grade 5 RP. Ueki *et al.* [20] reported that the incidence of RP ≥ Grade 3 after SBRT was significantly higher in patients with IP than those without IP (10.0% vs 1.5%; *P* = 0.02). However, the combination of pulmonary fibrosis and emphysema (CPFE), which is defined by the presence of emphysema and parenchymal fibrosis in the same patient, has a poor prognosis, similar to that of idiopathic pulmonary fibrosis [21]. Pulmonary resection also leads to unsatisfactory outcomes in patients with CPFE. A retrospective review of 41 patients with CPFE following pulmonary resection for lung cancer showed that the cumulative survival rates at 3 and 5 years were 53.6% and 36.9%, respectively, and that 6 of 41 patients (14.6%) developed postoperative IP exacerbation [22]. Therefore, we speculate that the prognosis of IP largely influences the survival of patients with combined COPD and IP. Careful consideration is necessary in all cases when performing SBRT in patients with COPD with IP (especially those presenting focal honeycomb lesions [23]). Additionally, the clinical benefits of SBRT should be compared with those of surgical treatment in these patients.

We believe that SBRT may become a therapeutic option for patients with inoperable early-stage NSCLC who are undergoing LTOT due to poor ventilatory function. However, this study has a few limitations. First, it was a retrospective single-center study with a small number of patients. Second, the diagnosis of the enrolled patients was not homogenous and included COPD alone in some patients and COPD with IP in others. Therefore, further prospective multicenter studies are necessary.

## FUNDING

Funding to pay the Open Access publication charges for this article was provided by Respiratory Disease Center in Yokohama City University Medical Center.
